# Trichiniasis

**Published:** 1875-06

**Authors:** 


### TRICHINIASIS.

An unknown friend sends us a copy of an Erie (Pa.) newspaper, containing the report of some cases of trichiniasis, which have lately occurred in that vicinity, and papers also come from Pittsburgh and Cincinnati containing similar matter. The symptoms of this very strange affection were, in these cases, at first supposed to indicate some other disease, as is generally the fact. We do not mean to impute to the attending physicians lack of skill in diagnosis; for it is not likely that any practitioner would detect, at first, the true nature of the disorder, unless he has been forewarned. The symptoms are, in some respects, like those attending the continued fevers; but a close examination will satisfy the observer of the absence of the characteristic signs of any special type of fever.

The primary manifestations of this strange affection are pain in the bowels and diarrhœa. Vomiting sometimes occurs, and there is always more or less fever; even inflammation of the peritoneum, with its attendant high febrile excitement, may exist. These abdominal symptoms are caused by the presence and movements of the worms—the *trichinæ.* These little intruders do not seem to amount to much individually. They are so very minute that their presence is shown by the microscope only. In a specimen of human muscle examined by Prof. Dalton of this city, the largest of the trichinæ were about one twentieth of an inch in length; while in a single-file procession of the smaller members of the family, more than seventy were included in the space of an inch. In some of the examinations there were found seven thousand to the cubic inch of muscle; and in other cases it was estimated that as many as 85,000 occupied a single cubic inch. While in the muscles the trichinæ remain quietly coiled up, each in his little cosy cyst. But as soon as they are taken into the stomach they become lively and multiply rapidly. Some microscopist declares that he has found, in half a pound of lean pork, enough of the trichinæ to produce in the stomach a brood of thirty millions.

A few days after eating meat containing these worms, we observe the symptoms of intestinal irritation. The young trichinæ are then lively and have begun to work their way through the walls of the stomach and bowels. It is these perforations of the mucous membrane, infinitesimal in size but in numbers countless, which are the cause of the pain, vomiting and diarrhœa. Not many days are required for the entire moving hosts to escape from the stomach, and then, when the irritation they have produced ceases, these symptoms pass away. The parasites make their permanent settlements in the muscles, and, strangely enough, select the *voluntary* muscles. Having arrived at their new homes they produce what are known as the secondary symptoms of the trichinal affection. Pain in the muscles, very like rheumatism, is the principal symptom. Muscles sometimes become contracted and cannot be relaxed without great pain. There is present some degree of constitutional disturbance, fever, &c. These symptoms continue several weeks; or until the worms have passed from active life and retired to their private cysts, there to coil themselves up and henceforth remain quiet and harmless. If the patient recovers from the intestinal disturbance, and if he has the constitutional vigor to bear the more general attack on the system during the secondary stage of the trichinal affection, he is safe.

It will be seen that we base our hopes of his eventual recovery, principally on his own powers of endurance and recuperation. As yet, medical art affords but little help in the way of *treatment.* It is possible that if the nature of the disease be detected during the period of the gastric and intestinal disturbance, some means may be used to destroy the trichinæ while yet in the stomach and bowels. But as these symptoms are caused by the perforation of the mucous membrane and other tissues of the alimentary canal, through which the verminous army pass in their march to the muscles, the advanced column will be beyond the reach of the longest-ranged vermifuge. There are no specifics for the constitutional affection. When the rheumatic and other symptoms indicate the presence of trichinæ in the muscles, then all that can be done is to treat the patient on "general principles." Relieve the pains, keep down the fever, and keep up the strength, by means that will readily suggest themselves to any physician, and hope for a favorable result.

Though the treatment is so uncertain and so little understood, yet the means of prevention are sure and simple. If the pork, and other food into which that meat enters as a constituent, is thoroughly cooked no trouble can result from trichinæ. The heat required for thorough cooking is sufficient to destroy the worms. Perhaps the most dangerous article of the pork kind is boiled ham, when *underdone.* People sometimes eat ham raw, or taste it raw when they wish to "sample" a specimen.

This should never be done. The smallest morsel may contain a few of the encysted trichinæ, and these few, when they get into the stomach, will produce a multitude. The mosaic prohibition of the use of the flesh of the unclean beast was authorized by divine wisdom. It would be well for humanity if all should heed, as do the faithful Jews, this law of the old dispensation. But if people will eat pork, we repeat, *let it be well cooked.*

